# Selenium hyperaccumulation offers protection from cell disruptor herbivores

**DOI:** 10.1186/1472-6785-10-19

**Published:** 2010-08-27

**Authors:** Colin F Quinn, John L Freeman, Ray JB Reynolds, Jennifer J Cappa, Sirine C Fakra, Matthew A Marcus, Stormy D Lindblom, Erin K Quinn, Lindsay E Bennett, Elizabeth AH Pilon-Smits

**Affiliations:** 1 Department of Biology, Colorado State University, Fort Collins, CO 80532, USA; 2 Agricultural Research Service, U.S. Department of Agriculture, Parlier, CA 93648, USA; 3 Advanced Light Source, Lawrence Berkeley National Laboratory, Berkeley, CA 94720, USA; 4 California State University Fresno, Center for Irrigation Technology, Fresno CA 93740, USA

## Abstract

**Background:**

Hyperaccumulation, the rare capacity of certain plant species to accumulate toxic trace elements to levels several orders of magnitude higher than other species growing on the same site, is thought to be an elemental defense mechanism against herbivores and pathogens. Previous research has shown that selenium (Se) hyperaccumulation protects plants from a variety of herbivores and pathogens. Selenium hyperaccumulating plants sequester Se in discrete locations in the leaf periphery, making them potentially more susceptible to some herbivore feeding modes than others. In this study we investigate the protective function of Se in the Se hyperaccumulators *Stanleya pinnata *and *Astragalus bisulcatus *against two cell disrupting herbivores, the western flower thrips (*Frankliniella occidentalis*) and the two-spotted spider mite (*Tetranychus urticae*).

**Results:**

*Astragalus bisulcatus *and *S. pinnata *with high Se concentrations (greater than 650 mg Se kg^-1^) were less subject to thrips herbivory than plants with low Se levels (less than 150 mg Se kg^-1^). Furthermore, in plants containing elevated Se levels, leaves with higher concentrations of Se suffered less herbivory than leaves with less Se. Spider mites also preferred to feed on low-Se *A. bisulcatus *and *S. pinnata *plants rather than high-Se plants. Spider mite populations on *A. bisulcatus *decreased after plants were given a higher concentration of Se. Interestingly, spider mites could colonize *A. bisulcatus *plants containing up to 200 mg Se kg^-1 ^dry weight, concentrations which are toxic to many other herbivores. Selenium distribution and speciation studies using micro-focused X-ray fluorescence (μXRF) mapping and Se K-edge X-ray absorption spectroscopy revealed that the spider mites accumulated primarily methylselenocysteine, the relatively non-toxic form of Se that is also the predominant form of Se in hyperaccumulators.

**Conclusions:**

This is the first reported study investigating the protective effect of hyperaccumulated Se against cell-disrupting herbivores. The finding that Se protected the two hyperaccumulator species from both cell disruptors lends further support to the elemental defense hypothesis and increases the number of herbivores and feeding modes against which Se has shown a protective effect. Because western flower thrips and two-spotted spider mites are widespread and economically important herbivores, the results from this study also have potential applications in agriculture or horticulture, and implications for the management of Se-rich crops.

## Background

For many organisms, including mammals and many species of bacteria and algae, selenium (Se) is an essential trace element [1]. These organisms contain selenoproteins, some of which destroy free radicals that damage DNA [2]. In humans, Se supplementation has been shown to reduce the chance of getting cancer, including the devastating widespread lung and prostate cancers [3,4]. In addition, Se plays an essential role in thyroid function [5]. While Se is essential for many organisms, there is a narrow margin between deficiency and toxicity levels. Selenium toxicity can be both acute and chronic. Acute Se toxicity leads to "blind staggers" in livestock; the symptoms include staggered walking, impaired vision, paralysis and sometimes death. Chronic Se poisoning leads to hair and nail loss, fatigue, nausea and eventually death [6].

Selenium has no known essential function for higher plants, and elevated levels of Se are toxic to most plants [7]. This toxicity is due to the chemical similarity of Se and sulfur (S). Most plants inadvertently assimilate Se into proteins, leading to toxicity [1]. A few plant species have evolved to accumulate unusually large amounts of Se, as much as 1%, or 10,000 mg Se kg^-1 ^dry weight (DW) [8,9]. These unique plants are called Se hyperaccumulators and they avoid Se poisoning by methylating SeCys to form methylselenocysteine (MeSeCys), which is relatively non-toxic because it does not get incorporated into proteins [10].

Hyperaccumulation is a phenomenon where plants accumulate particular elements to levels several orders of magnitude higher than other plant species growing on the same substrate [11]. Some other elements besides Se that can be hyperaccumulated by plants include aluminum (Al), arsenic (As), cadmium (Cd), manganese (Mn), nickel (Ni) and zinc (Zn) [12]. Feist and Parker [13] defined Se hyperaccumulation as plants that contain more than 1,000 mg Se kg^-1 ^DW. Most research investigating the functional significance of hyperaccumulation has focused on and lent support to the elemental defense hypothesis, which states that plants have evolved to hyperaccumulate these various toxic elements as protection against herbivore and pathogen attacks [14]. Hyperaccumulated As, Cd, Ni, Zn and Se all have been shown to protect plants from herbivores and/or pathogens [15-19].

To date, Se hyperaccumulation has been shown to protect plants from a mammalian herbivore, the black-tailed prairie dog (*Cynomys ludovicianus*), as well as from several arthropod herbivores and two fungal pathogens [20-23]. Additionally, Se hyperaccumulating plants harbored fewer arthropods and arthropod species than comparable non Se hyperaccumulators growing in the same, seleniferous habitat [24]. Moreover, Se hyperaccumulating plants sequester Se in organs and tissues that are most susceptible to herbivore attack. For example, the Se in hyperaccumulator *Astragalus bisulcatus *(two-grooved milk vetch) is predominantly present in the leaf hairs, and *Stanleya pinnata *(Prince's plume), another Se hyperaccumulator, sequesters Se in epidermal cells in the leaf margins [9]. This uneven distribution of Se, which leaves some areas of the plant with lower concentrations of Se than others, may allow some herbivores, depending on their feeding mode, to avoid this elemental defense. Indeed, some herbivore species were found living on Se hyperaccumulating plants in the field and apparently were feeding on the Se-rich plant material, in view of the fact that they contained higher Se concentrations than individuals collected from non-hyperaccumulators [24]. Thus, it is important to investigate the effect of feeding mode on the herbivores' ability to feed on Se hyperaccumulating plants. The effect of feeding mode on herbivore susceptibility to hyperaccumulated elements is illustrated by the study by Jhee et al. [25] who found that the Ni hyperaccumulator *Streptanthus polygaloides *was protected from folivore herbivores but not vascular feeding herbivores. In addition, the Zn hyperaccumulator *Thlaspi caerulescens *was not protected from snail herbivory [26]

This study investigates the protective effect of Se hyperaccumulation against cell disruptor herbivore species, specifically the two-spotted spider mite (*Tetranychus urticae*) and western flower thrips (*Frankliniella occidentalis*). Both herbivores have been observed feeding on *A. bisulcatus *and *S. pinnata *in the greenhouse and both feed by piercing the cell surface with their mouthparts and sucking out the cell contents [27]. This study, the first to examine cell disruptor herbivores' sensitivity to Se hyperaccumulation, is ecologically relevant because both of these herbivores share habitats with Se hyperaccumulating plants [13,28]. Interestingly, western flower thrips and many Se hyperaccumulating plant species are native to the western United States and protection against thrips herbivory may have contributed to the evolution of Se hyperaccumulation. Both herbivores are also ecologically important pests. Two-spotted spider mites can have devastating effects on crop yields worldwide [29]. Outbreaks often occur after pesticide application inadvertently kills their predators [30]. Western flower thrips are native to the Western United States, but have been reported on all continents except Asia and Antarctica [31]. Through a combination of their herbivory and their notorious ability to transfer disease and develop pesticide resistance, western flower thrips can significantly reduce crop yields [32,33]. In this study we report a significant effect of plant Se accumulation on the response of two Se hyperaccumulators (*A. bisulcatus *and *S. pinnata*) to both cell disruptors, the western flower thrips and the two-spotted spider mite.

## Methods

### Plant material

Seeds of *A. bisulcatus *were obtained from plants growing at Pine Ridge Natural Area in Fort Collins, CO, USA (40°32.70N, 105°07.87W). Pine Ridge Natural Area is a seleniferous habitat; the population of *A. bisulcatus *from which seeds were collected accumulates up to 10,000 mg Se kg^-1 ^[34]. Seed germination and growth followed an arid western plant growth protocol previously used for Se hyperaccumulating plants and described by Sors et al. [35]. Plants were grown on pre-washed Turface MVP (Profile Products LLC, Buffalo Grove, IL) in 25 cm diameter pots in greenhouse conditions (24/20°C day/night, 16-h photoperiod, 300 μmol m^-2 ^sec^-1 ^photosynthetic photon flux). Three weeks after germination half of the plants received high-Se fertilizer treatments, 1 g of fertilizer (Miracle-Gro Excel, 15:5:15 Cal-Mag, The Scotts Co., Marysville, OH) per liter of water combined with 20 μM Na_2_SeO_4_, while the other half received low-Se fertilizer treatments, 1 g of fertilizer per liter of water with 2 μM Na_2_SeO_4_, three times a week. After 20 weeks of growth plants were used for thrips and spider mite experiments as described below.

*Stanleya pinnata *seeds were obtained from Western Native Seed (Coaldale, CO, USA) and plants were grown from seed in pre-washed Turface MVP. Thirty-six plants were grown in a growth room (24°C/20°C, 12 h/12 h light/dark, 120 μmol m^-2 ^s^-1 ^photosynthetic photon flux): 10 weeks after germination half of the plants were watered twice a week for 50 weeks with 1 g of fertilizer (Miracle-Gro Excel, 15:5:15 Cal-Mag, The Scotts Co., Marysville, OH) per liter of water and 20 μM Na_2_SeO_4_, the other half were watered with 1 g of fertilizer per liter of water as a control. Plants were used for thrips and spider mite experiments as described below.

### Effects of Se on herbivory of *A. bisulcatus *by thrips

To investigate thrips toxicity to Se and their preference to feed on high- or low- Se plants, both non-choice and choice experiments were conducted. For non-choice experiments high- and low-Se *A. bisulcatus *were infected with western flower thrips by placing three excised *A. bisulcatus *leaves previously harboring large populations of thrips on each plant. Thrips were initially acquired from *A. bisulcatus *plants growing in a greenhouse and suffering thrips infestation. Two high and low-Se plants were then placed in separate 20 g glass tanks that were kept in 24/20°C day/night, 16-h photoperiod, 300 μmol m^-2 ^sec^-1 ^photosynthetic photon flux and watered 3 times a week either with 20 μM Na_2_SeO_4 _or without Se. Tanks were covered with 0.2 mm^2 ^nylon mesh tops to prevent thrips transfer while still allowing gas exchange. For choice experiments plants were infected with thrips as described above and a high- and a low-Se plant were placed in the same glass tank. After three weeks of herbivory the percentage of young (mature leaves from the top five nodes), medium (leaves from middle nodes) and old (leaves from the bottom 3 nodes) leaves and the percentage of leaflets per leaf with visual signs of thrips herbivory were calculated on each plant. Non-choice experiments were repeated 6 times and choice experiments were repeated 4 times for high-Se and 4 times for low-Se treatments. Selenium concentrations for young, medium and old leaves were measured as described below.

### Effects of Se on herbivory of *A. bisulcatus *by spider mites

Spider mite non-choice and choice experiments were also conducted using *A. bisulcatus*. For non-choice experiments 10 high- or low-Se plants grown as described above were placed in 20 L glass tanks that were placed in 24/20°C day/night, 16-h photoperiod and 300 μmol m^-2 ^sec^-1 ^photosynthetic photon flux and covered with 0.2 mm^2 ^mesh. Plants were watered either with 20 μM Na_2_SeO_4 _or without Se 3 times weekly. Each plant was inoculated with 100 spider mites that were collected from *A. bisulcatus *plants with high spider mite populations. The number of spider mites on each plant was counted after 7, 14 and 21 days and the percent of the population change was calculated for each plant. For choice experiments spider mites were given a choice to feed on high- or low-Se plants. One high- and one low-Se *A. bisulcatus *plant grown as described above was placed in a tank and 100 spider mites were placed on each plant. The number of spider mites on each plant was counted after 7, 14 and 21 days and percent population change was calculated. This experiment was repeated 7 times. Leaf Se concentrations of youngest mature leaves were compared between high- and low-Se plants as described below.

In addition to choice and non-choice experiments, low-Se *A. bisulcatus *pre-infected with spider mites were provided with Se to determine if adding Se reduces established populations of spider mites. At the onset of the experiment low-Se *A. bisulcatus *plants that were being treated with 2 μm Se were infected with large spider mite populations. For three weeks eight of the plants were provided with 40 μm Se three times a week while eight others were provided with water as a control. The percent population change in spider mite population was recorded after 7, 14 and 21 days. Leaf Se concentration of the youngest mature leaves were measured before and after the experiment.

Spider mites from high-Se *A. bisulcatus *plants used in non-choice experiments were collected and analyzed for Se speciation. Samples were washed and flash-frozen using liquid nitrogen. Samples were kept frozen at -80°C to prevent Se metabolism, and Se speciation was determined using XANES as described by Marcus et al. [36], using known selenocompounds as standards.

### Se speciation, X-ray microprobe measurements

Spider mites from high-Se *A. bisulcatus *plants used in non-choice experiments were collected and analyzed for Se speciation. Samples were washed and flash-frozen using liquid nitrogen. Samples were kept frozen to prevent Se metabolism, and Se speciation was determined using XANES as described earlier [21,36], using well characterized selenocompounds as standards.

### Effects of Se on herbivory of *S. pinnata *by thrips

*Stanleya pinnata*, another Se hyperaccumulating species, was also used to determine if Se protects against cell disrupting herbivores. Thrips were given a choice to feed on either high- or low-Se *S. pinnata*. Eighteen high-Se and 18 low-Se plants grown as described above were intermixed and placed in a growing room (12 h/12 h light/dark, 120 μmol m^-2 ^s^-1 ^photosynthetic photon flux) heavily infested with thrips. Plants were watered either with 20 μM Na_2_SeO_4 _or without Se 3 times weekly. After 4 weeks of being exposed to thrips herbivory the percentage of leaves with visual signs of thrips herbivory was compared between plants with and without Se. In addition, since within one *S. pinnata *plant Se is unevenly distributed we determined Se concentration of leaves with thrips herbivory and leaves without thrips herbivory from the same high-Se plant. For six of the *S. pinnata *plants treated with Se two similar-aged leaves per plant, one with herbivory and one without herbivory, were collected and analyzed for elemental concentrations using ICP-AES, as described below. To determine the variation in Se concentration of leaf age in plants not suffering herbivory, three leaves from consecutive nodes on the same high-Se *S. pinnata *plants were tested for Se concentration. This was repeated for 6 plants.

### Effects of Se on herbivory of *S. pinnata *by spider mites

Ten high-Se and nine low-Se *S. pinnata *grown as described above were interspersed in a 50 cm × 50 cm area on a greenhouse bench. Each plant was watered 3 times weekly either with 20 μM Na_2_SeO_4 _or without Se and was infected with spider mites by placing three leaves from other plants that harbored high concentrations of spider mites on the center of each plant. Spider mites were allowed to forage for two weeks, and herbivory was then scored by counting the number of leaves on each plant with and without visual signs of spider mite herbivory. The youngest mature leaves were collected from each plant and analyzed for Se concentration.

### Elemental analysis

Elemental concentrations in leaves were determined by digesting approximately 100 mg DW of leaf material in 1 ml of nitric acid as described by Zarcinas et al. [37]. Using distilled water the samples were diluted to 10 ml and elemental concentrations were determined using Inductively Coupled Plasma Atomic Emission Spectrometry (ICP-AES) as described by Fassel [38].

### Data analysis

The software package JMP-IN (3.2.6, SAS Institute, Cary, NC) was used for all data analysis. Student's t-test was used to compare differences in herbivory between high-Se and low-Se plants and to compare elemental concentrations of leaf samples.

## Results

### Effects of Se on herbivory of *A. bisulcatus *by thrips

**Figure 1:**
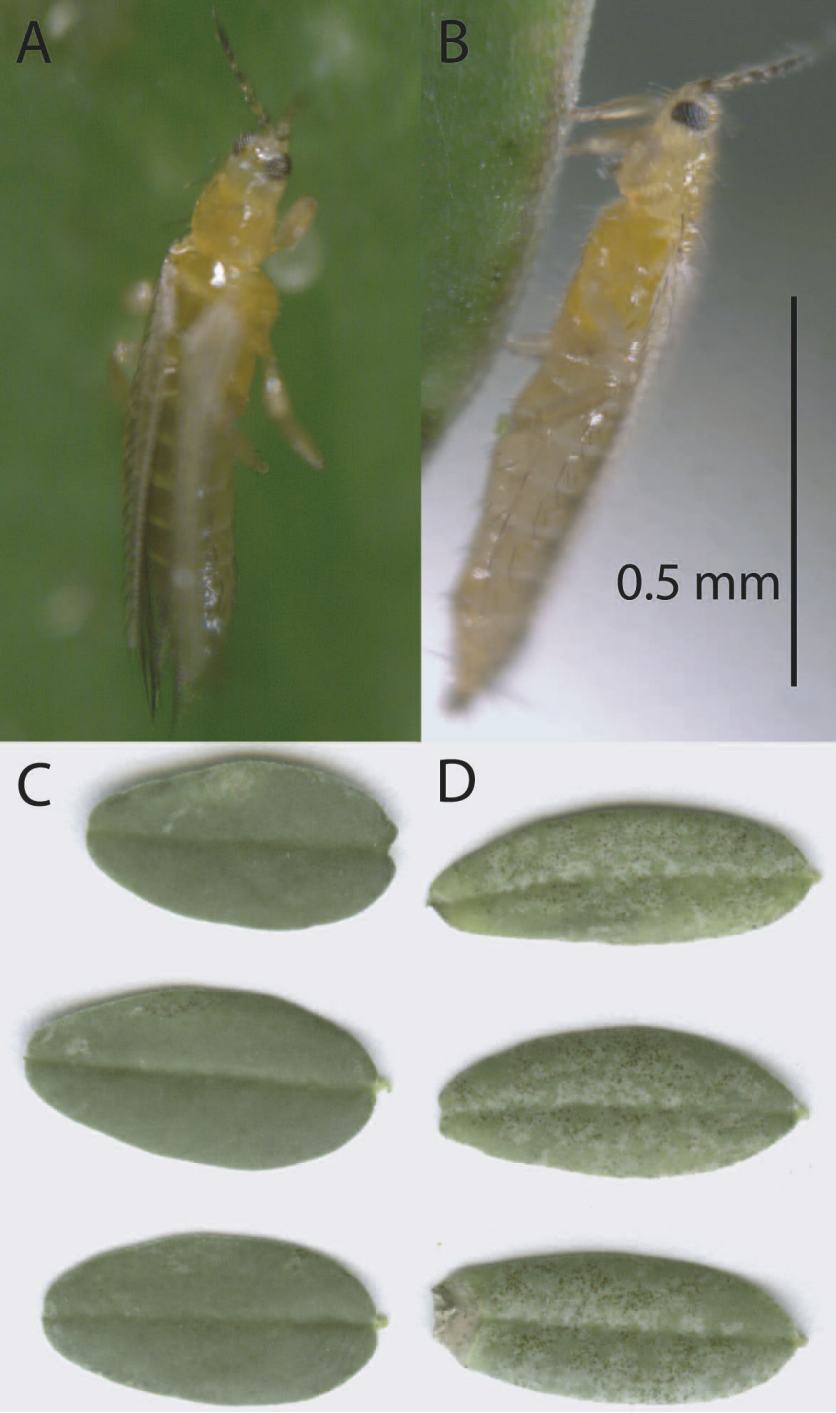
**Western flower thrips feeding on *A. bisulcatus***. A, B: Thrips piercing A. bisulcatus leaflets, as viewed from the top (A) and side (B).  C, D: Representative leaflets from high-Se (C) and low-Se (D) *A. bisulcatus* plants after exposure to thrips herbivory. Herbivory damage is apparent as white patches with black spots.

**Figure 2:**
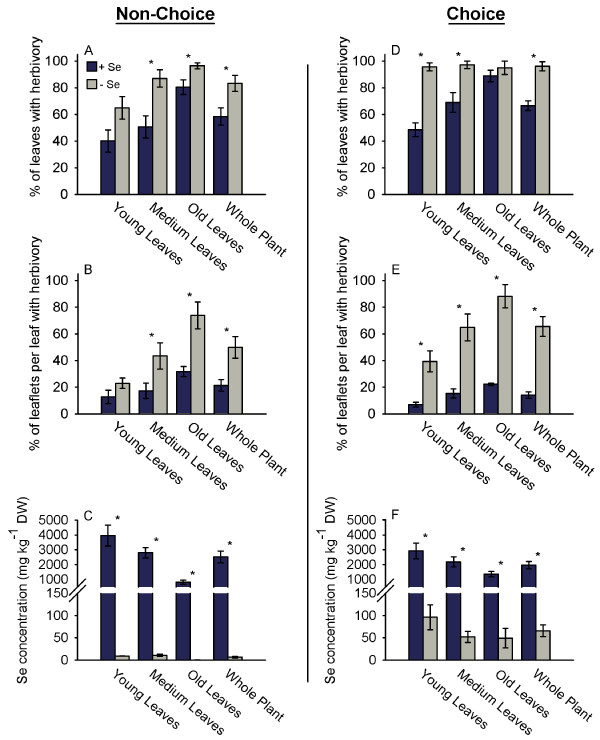
**Selenium reduces thrips herbivory to A. bisulcatus in both choice and non-choice experiments.** A-C: Thrips non-choice feeding experiment where thrips were offered only high-Se or low-Se plants.  Herbivory was quantified as the percentage of entire A. bisulcatus young, medium and old leaves that showed herbivory (A) and as the percentage of leaflets per leaf that showed herbivory (B). The leaf Se concentration of the high-Se and low-Se plants used in the non-choice studies is shown in panel C.  D-F: Thrips choice feeding experiments where thrips were provided with a choice between high-Se and low-Se plants. Herbivory was quantified as the percentage of A. bisulcatus young, medium and old leaves that showed herbivory (D) and as the percentage of leaflets per leaf (E) that suffered herbivory.  The leaf Se concentration of the plants used in the choice study is shown in panel F. Values are means +/- SE.  An asterisk above a pair of bars represents a significant difference between the high-Se and low-Se treatments (t-tests, α = 0.05, n = 6 for both high-Se and low-Se non-choice experiments, n = 4 for choice experiments).

To investigate if the Se hyperaccumulator *A. bisulcatus *was protected from western flower thrips, both choice and non-choice studies were conducted with plants containing high and low concentrations of Se (the thrips are displayed in Figure 1A, B; high- and low-Se leaflets exposed to thrips herbivory are shown in Figure 1C, D). The non-choice experiments revealed that the fraction of leaves with herbivory was significantly less for high-Se plants than low-Se plants (p = 0.018, t = -2.832, n = 6 for both high- and low-Se experiments), and that younger leaves suffered less herbivory than older leaves from both high- and low-Se plants (Figure 2A). In addition, fewer leaflets per leaf suffered thrips herbivory on high-Se than low-Se plants (Figure 2B; p = 0.011, t = -3.095, n = 6 for both high- and low-Se experiments). Young leaves from high-Se plants contained roughly 1.5-fold and 5-fold higher Se concentrations than medium-aged and old leaves (respectively) of the same plants, ranging from 3,945 mg Se kg^-1 ^for young leaves and 812 mg Se kg^-1 ^for old leaves, while leaves from low-Se plants did not reach above 11 mg Se kg^-1 ^(Figure 2C).

When thrips were given a choice to feed on high- or low-Se plants they showed a significant preference to colonize low-Se plants. In these choice experiments low-Se leaves and leaflets suffered more herbivory than high-Se leaves and leaflets (Figure 2D p = 0.001, t = -5.926; Figure 2E, p < 0.001, t = -6.443 n = 4 pairs of high- and low-Se plants). On high-Se plants young leaves suffered less herbivory than old leaves (Figure 2D, p = 0.001, t = 5.913, n = 4 pairs of high- and low-Se plants). Similar to what was found for plants used in the non-choice thrips experiments, young leaves of the high-Se plants contained more Se than old leaves, 3,000 mg Se kg^-1 ^compared to 1,350 mg Se kg^-1^, respectively (Figure 2F). While in the choice study high-Se plants had many fold higher Se concentrations than low-Se plants, leaves from low-Se plants also contained around 100 mg Se kg^-1 ^DW in young leaves and approximately 50 mg Se kg^-1 ^DW in medium-aged and old leaves (Figure 2F).

### Effects of Se on herbivory of *A. bisulcatus *by spider mites

**Figure 3:**
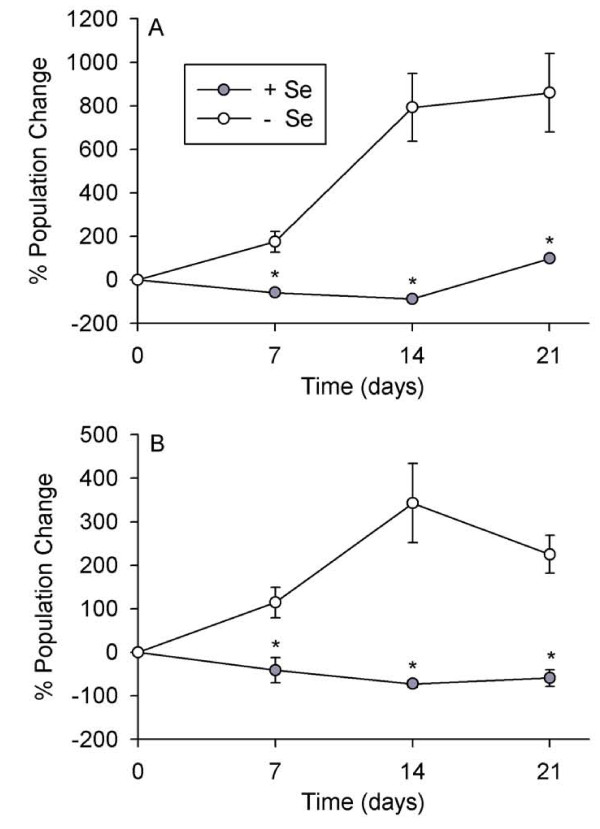
**Growth of spider mite populations feeding on high-Se or low-Se *A. bisulcatus* over the course of a non-choice feeding study (A) and a choice feeding study (B).**  Values are means +/- SE. An asterisk between data points in the non-choice or choice feeding experiments represents a significant difference between high-Se and low-Se plants (t-tests, α = 0.05).

Non-choice and choice experiments were conducted to determine if Se effectively protected *A. bisulcatus *from another cell disruptor herbivore, the two-spotted spider mite. During the non-choice study spider mite populations only gradually increased in size on high-Se plants, whereas plants pre-treated with a low Se concentration showed an 800% spider mite population growth rate over three weeks (Figure 3A; p < 0.001, t = 5.306, n = 10 high- and 10 low-Se plants). When spider mites were given a choice to feed on high- or low-Se plants they preferred low-Se plants. The protective effect of Se was already detectable after one week, as populations of spider mites on high-Se plants decreased in size over time while populations on low-Se plants increased by over 200% after three weeks (Figure 3B; p < 0.001, t = 6.004, n = 7 high- and 7 low-Se plants). High-Se plants contained over 2,200 mg Se kg^-1 ^DW and low-Se plants contained 110 mg Se kg^-1 ^DW (p = 0.009, t = -4.792, n = 3 high- and 3 low-Se plants).

**Figure 4:**
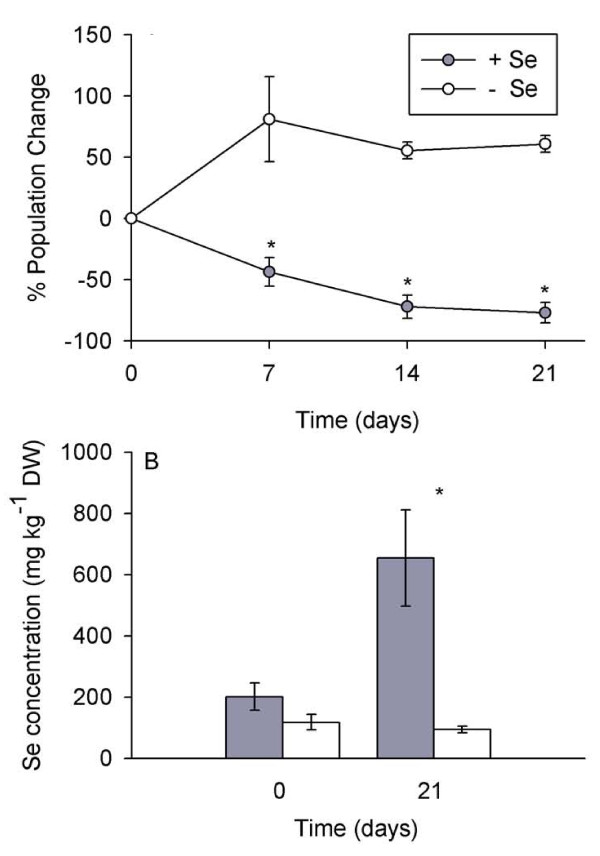
**Selenium added to spider mite-populated *A. bisulcatus* plants reduced spider mite population growth.** A: Percent population change of established spider mite populations on *A. bisulcatus* over the course of a 3-week high-Se or low-Se treatment.  B: Selenium concentration of plants at the beginning and end of the experiment.  Values show means +/- SE.  An asterisk between data points (A) or bars (B) represents a significant difference between the high- and low-Se treatments (t-test, α = 0.05, n = 10 for non-choice experiments, n = 7 for choice experiments).

Another experiment was conducted to investigate the effect of adding Se to *A. bisulcatus *pre-infested with spider mites. Half of the plants infested with spider mites was treated with Se and the other half was given water as a control. After seven days the Se treatment had resulted in a 50% reduction in the population of spider mites; in contrast, the population of spider mites on plants not treated with Se increased by 50% during the same time period (Figure 4A, p = 0.004, t = 3.416, n = 8 high- and 8 low-Se plants). Three weeks after the start of the Se treatment the spider mite populations on the high-Se plants had decreased by almost 80% while the populations of spider mites on low-Se plants still showed an increase of 50% (Figure 4A, p < 0.001, t = 12.807, n = 8 high- and 8 low-Se plants). Prior to conducting the experiment, all *A. bisulcatus *plants contained between 100 - 200 mg Se kg^-1 ^DW. After the three-week experiment the high-Se plants contained almost 800 mg Se kg^-1 ^DW and the low-Se plants contained 100 mg Se kg^-1 ^(Figure 4B, p = 0.010, t = 2.870, n = 8 high- and 8 low-Se plants).

**Figure 5:**
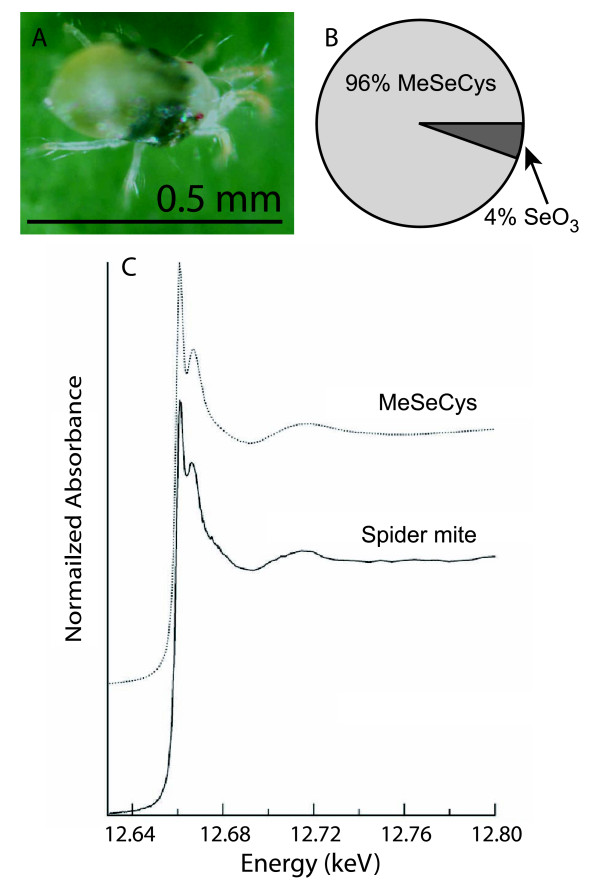
**Selenium speciation in spider mites collected from Se-rich *A. bisulcatus* plants.** X-ray analysis of near-edge spectra (XANES) revealed that two-spotted spider mites collected from Se-rich *A. bisulcatus* plants (shown in panel A) contained primarily methylselenocysteine (B, C).  The XANES Se spectra of the spider mites and of the methylselenocysteine standard compound are shown in panel C.

Since the spider mites appeared to tolerate plant Se concentrations up to 150 mg Se kg^-1 ^we collected spider mites off Se-treated plants to investigate the mechanism of their relatively high Se tolerance at the biochemical level. Selenium speciation studies using Se K-edge (X-ray absorption near-edge structure (XANES)) spectroscopy and least square linear combination fitting (LCF) of the XANES spectra using standard compounds revealed that spider mites store Se primarily as an organic C-Se-C form similar to methylselenocysteine (MeSeCys) (Figure 5A-C).

### Effects of Se on herbivory of *S. pinnata *by thrips

**Figure 6:**
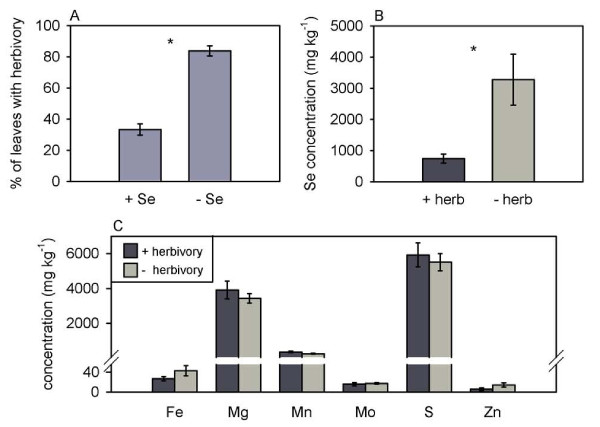
**Selenium prevents thrips herbivory to *S. pinnata* in both choice and non-choice studies.** A: Choice feeding experiment quantifying thrips herbivory of *S. pinnata* plants treated with or without Se, quantified as percentage of leaves per plant showing herbivory.  B: Selenium concentration in plants treated with Se, comparing leaves that experienced thrips herbivory with leaves showing no herbivory.  C: Elemental concentration of Fe, Mg, Mn, Mo, S and Zn in plants treated with Se, comparing leaves that experienced thrips herbivory with leaves showing no herbivory. Values are means +/- SE.  An asterisk between a pair of bars represents a significant difference between the two treatments (t-tests, α = 0.05, n = 18).

**Figure 7:**
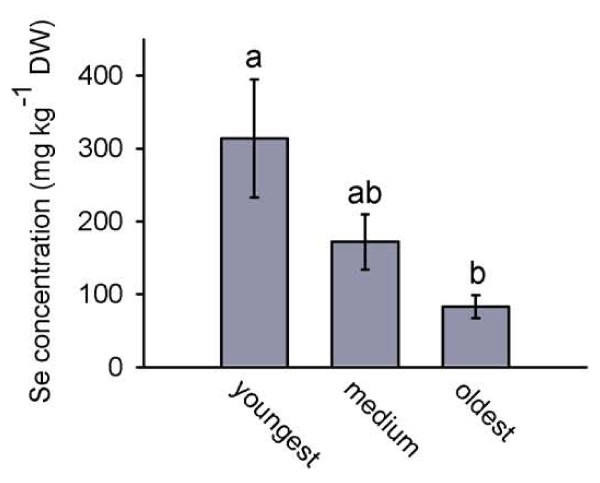
***S. pinnata* plants show an age-related difference in leaf Se concentration.** Shown is Se concentration in three young leaves from consecutive nodes. Values are means +/- SE. (Tukey-Kramer test, α = 0.05, n = 6).

To further investigate if Se hyperaccumulators are protected from cell disrupting herbivores we used another Se hyperaccumulating plants species, *S. pinnata*, and again used thrips in a choice herbivory experiment. The thrips preferred to feed on *S. pinnata *plants without Se when given a choice between high- and low-Se plants (Figure 6A, p < 0.001, t = -10.333, n = 18 high- and 18 low-Se plants). Within the Se-treated plants, leaves with elevated Se suffered less herbivory than similar-aged leaves on the same plants with lower Se levels (Figure 6B, p = 0.012, t = -3.056, n = 6 high- and 6 low-Se plants). The leaves that were compared had similar concentrations of other elements beside Se (Figure 6C). To determine if the difference in Se concentration found in each pair of leaves was a response to herbivory or rather a leaf age-related difference in Se concentration to which the herbivore responded, Se concentration as a function of leaf age was investigated in more detail in plants without herbivory. Three leaves from consecutive nodes on each of six high-Se plants were analyzed for Se. The youngest of the three leaves contained a higher Se concentration than the oldest-aged leaf (Figure 7).

### Effects of Se on herbivory of *S. pinnata *by spider mites

Spider mites were given a choice to feed on either high-Se or low-Se *S. pinnata *to determine if elevated Se concentrations protected *S. pinnata *from spider mite herbivory. On plants with elevated Se only 35% of leaves suffered spider mite herbivory while over 75% of leaves from low-Se plants suffered spider mite herbivory (p = 0.002, t = 3.617, n = 10 high and 9 low-Se plants). High-Se plants contained 420 mg Se kg^-1 ^compared to low Se plants, which only had 50 mg Se kg^-1 ^(p = 0.007, t = -3.078, n = 10 high and 9 low-Se plants).

## Discussion

These results expand on previous studies investigating the functional significance of Se hyperaccumulation. The earlier studies have shown that elevated Se can protect plants from arthropod folivore herbivores (grasshoppers, caterpillars), grazing mammalian herbivores (prairie dogs), phloem-feeding arthropods (aphids) and leaf and stem/root fungal pathogens [18,20,21,23]. This is the first study to show that Se protects hyperaccumulating plants from cell disrupting herbivores. This study provides evidence that two Se hyperaccumulating species, *S. pinnata *and *A. bisulcatus*, are protected against two ecologically relevant and economically important cell disruptor herbivores, the two-spotted spider mite and the western flower thrips, only when containing elevated Se concentrations. The non-choice studies showed that high-Se plants suffered less spider mite and thrips herbivory. The choice studies demonstrated that spider mites and thrips preferred low-Se *A. bisulcatus *and *S. pinnata *plants over high-Se plants. Furthermore, within a single plant, low-Se leaves suffered more thrips herbivory than high-Se leaves. Studies using *A. bisulcatus *showed that thrips preferred to feed on older leaves, which contained less Se. Studies with *S. pinnata *showed that leaves with high concentrations of Se suffered less thrips and spider mite herbivory than low-Se leaves and that younger leaves, even when only one node apart, had higher Se concentrations than older leaves. Those results suggest that these plants preferentially sequester Se in their younger leaves, which may be more valuable than older leaves because of higher photosynthesis rates [39], and in doing so are successful in protecting what may be considered their more valuable parts against these herbivores. Interestingly, in non-choice studies we did not find significant differences in thrips herbivory damage on young leaves of high- and low-Se plants but did find more thrips herbivory on low-Se medium aged and old leaves compared to high-Se medium aged and old leaves (Figure 2A,B). It is possibly that these young leaves contain more nutrients than older leaves, which is true in other plant species [40], and therefore are a more attractive food source for herbivores. The results of this study lend further support to the hypothesis that Se hyperaccumulation serves as protection against herbivore attacks, and expands the list of herbivores against which Se is effective.

Herbivore feeding mode can be an important factor in plant-herbivore interactions. It is likely that some herbivores can circumvent plant defenses, including elemental defense, as a result of feeding modes [41,42] and that different hyperaccumulating plants are protected from different groups of herbivores. For example, Ni hyperaccumulation does not appear to protect plants from xylem and phloem feeding herbivores [25], while elevated Se, even at concentrations as low as 10 mg Se kg^-1 ^DW, can protect plants from the phloem-feeding green peach aphid [18]. Studies investigating Se distribution in Se hyperaccumulating plants suggests that they are better protected from some feeding modes than others. Leaves of *S. pinnata *sequester Se in the periphery of the leaves, in the epidermal cell layer, which is expected to be particularly effective against many folivores, like grasshoppers and caterpillars [9]. *Astragalus bisulcatus *leaves sequester Se in trichomes, which may act as an initial defense mechanism against a variety of feeding types [9].

Interestingly, it appears that spider mites can tolerate plant Se concentrations in hyperaccumulators up to ~150 mg Se kg^-1 ^DW, concentrations that are toxic to many other herbivores (Figure 4A, B) [18,22]. Selenium speciation studies revealed that the spider mites accumulated an organic form of Se indistinguishable from MeSeCys (Figure 5B, C). This form of Se is less toxic than many other forms of Se because it is not incorporated into proteins [10]. The same form of Se was found in Se hyperaccumulator plants as well as in Se-tolerant herbivores found feeding on hyperaccumulators [21]. If the spider mites accumulate MeSeCys as well, this may contribute to their tolerance of relatively high concentrations of Se. It should be noted, however, that XANES does not effectively distinguish between various C-Se-C compounds, including MeSeCys, selenomethionine, and Se-cystathionine [9] and therefore it is possible that the mites accumulated a more toxic form of Se, or a mixture of these organic selenocompounds. This would explain why, at higher Se levels (around 420 mg Se kg^-1 ^DW for *S. pinnata *and 800 mg Se kg^-1 ^DW for *A. bisulcatus*) Se effectively protected the plants, even against spider mites.

These results have important implications for managing Se-rich agricultural or natural areas and Se phytoremediation or biofortification crops. Crops in seleniferous habitats and plants used for Se phytoremediation often do not accumulate more than 150 mg Se kg^-1 ^[43]. While these plants may be protected by their low Se levels from folivore arthropods, they may still be susceptible to spider mite herbivory. On the other hand, Se hyperaccumulating plants, which typically contain more than 1,000 mg Se kg^-1 ^DW [24], likely are protected against both folivores and spider mites. This combined protective effect of Se accumulation against such a wide variety of herbivores may have been an important driving force for the evolution of Se hyperaccumulation.

## Conclusions

Herbivores with different feeding modes may respond differently to hyperaccumulation in plants, as was suggested by Jhee et al. [25]. Because Se hyperaccumulating plants preferentially allocate Se to specific locations they may leave other locations vulnerable to herbivore attacks. This study shows that Se hyperaccumulating plants are protected from two economically important cell disrupting herbivores. The western flower thrips is considered a major pest because it is known to feed on plants in over 62 different families including many crop species [44], they effectively transfer viruses to crop species [45] and they rapidly develop pesticide resistance [46,47]. Two-spotted spider mites are also known to target many crops, such as fruit trees and vegetables, and can also develop resistance to pesticides [48]. The results of this study provide support for the elemental defense hypothesis and have implications for management of seleniferous habitats and Se phytoremediation. Selenium may act as a natural pesticide in Se-rich crops and plants used for Se phytoremediation in areas such as the western United States, where two-spotted spidermites, western flower thrips and Se hyperaccumulators all occur and where Se-rich agriculture is present. The observed avoidance of Se-rich plants by herbivores may also reduce the probability of Se movement and bioconcentration in the food chain. However, the ability of spider mites to tolerate 150 mg Se kg^-1 ^may allow transfer of Se into higher trophic levels, which is an area that needs to be further investigated.

## Authors' contributions

CFQ, JLF, RJBR and EAHPS conceived and coordinated the experiments in this study. SCF performed μXRF and μXAS data analyses. All authors assisted with the experiments. CFQ, JLF and EAHPS drafted the manuscript. All authors read and approved the final manuscript.

## Acknowledgements

This research was supported by grants #IOB-0444471 and #IOS-0817748 from the National Science Foundation to EAHPS. We thank David Steingraeber, Arathi Seshadri and Mark Paschke for helpful comments on the manuscript. We would also like to thank 2 anonymous reviewers for helpful comments that improved the manuscript. The Advanced Light Source is supported by the Office of Science, Basic Energy Sciences, Division of Materials Science of the U.S. Department of Energy (DE-AC02-05CH11231).
